# Severe bilateral ischemic-reperfusion renal injury: hyperacute and acute changes in apparent diffusion coefficient, T1, and T2 mapping with immunohistochemical correlations

**DOI:** 10.1038/s41598-017-01895-x

**Published:** 2017-05-11

**Authors:** Sheung-Fat Ko, Hon-Kan Yip, Yen-Yi Zhen, Chia-Chang Lee, Chen-Chang Lee, Shyh-Jer Huang, Chung-Cheng Huang, Shu-Hang Ng, Jui-Wei Lin

**Affiliations:** 1 Department of Radiology, Kaohsiung Chang Gung Memorial Hospital and Chang Gung University College of Medicine, Kaohsiung, 833 Taiwan; 2 Department of Cardiology, Kaohsiung Chang Gung Memorial Hospital and Chang Gung University College of Medicine, Kaohsiung, 833 Taiwan; 3 Department of Medical Research, Kaohsiung Chang Gung Memorial Hospital and Chang Gung University College of Medicine, Kaohsiung, 833 Taiwan; 4 Department of Pathology, Kaohsiung Chang Gung Memorial Hospital and Chang Gung University College of Medicine, Kaohsiung, 833 Taiwan; 5 Department of Biomedical Engineering, I-Shou University, Kaohsiung, 833 Taiwan

## Abstract

The aim of this study was to investigate the hyperacute and acute changes in apparent diffusion coefficient (ADC), T1, and T2 mapping in rat kidneys after severe bilateral renal ischemic-reperfusion injury (IRI). After baseline MRI, 24 Spraque-Dawley rats with renal IRI were divided equally as group 1 (post-IRI MRI at 6 hours, days 1, 3, and 7) and groups 2, 3, and 4 (post-IRI MRI at 6 hours; 6 hours and day 1; 6 hours, days 1 and 3, respectively), while six other rats without IRI (group 5) were used as sham control. ADC, T1, and T2 values of the cortex and outer and inner stripes of outer medulla (OSOM and ISOM), and immunohistochemical studies assessing monocyte chemoattractant protein-1 (MCP-1), CD68+ cells, tubular cast formation, and collagen deposition in three zones at different time points were evaluated. Significantly reduced ADCs in OSOM and ISOM are noninvasive biomarkers denoting hyperacute damages after IRI. Linear regression analysis revealed a significant inverse correlation between 6-hour/baseline ADC ratios and MCP-1 staining (P < 0.001, r^2^ = 0.738). ADC, T1, and T2 values are useful for assessing variable IRI changes in different layers depending on underlying microstructural and histopathological changes at different time points.

## Introduction

Acute kidney injury (AKI) is common in hospitalized patients with increased risk of mortality^1^. There has been a substantial increase in the incidence of AKI in the recent two decades, and the population incidence of AKI necessitated renal replacement therapy is approximately 200–300 per million population per year^1, 2^. Various factors with significantly increased risks of AKI have been reported including old age, diabetes, hypertension, high baseline creatinine, heart failure, sepsis, use of nephrotoxic drugs or vasopressors, high risk or emergency surgery, use of intra-aortic balloon pump, and long term cardiopulmonary bypass pump^2, 3^. Recently, several AKI biomarkers such as neutrophil gelatinase-associated lipocalin, interleukin-18, L-type fatty acid-binding protein, N-acetyl-β-D-glucosaminidase, and kidney injury molecule have been reported^4^. However, the clinical usefulness of these biomarkers in the treatment decision-making process and differentiation of the causes of AKI are controversial^4, 5^. Conventional methods like serum creatinine, glomerular filtration rate, and urine output may be used but these are not reliable indicators of kidney damage^6^.

Despite the various risk factors of AKI that have been reported, renal tissue hypoxia or ischemic-reperfusion injury (IRI) is closely related to the pathophysiology of various initiating insults^3, 6–8^. Renal ultrasound is a common tool for the evaluation of the kidneys, but it is not sensitive for assessing renal IRI or AKI^9^. Various functional magnetic resonance imaging (MRI) techniques have been applied for assessing AKI^10–15^. Arterial spin labeling and dynamic contrast-enhanced magnetic resonance imaging are equally able to detect abnormal perfusion in AKI^11, 12^. Blood oxygen level-dependent imaging for measuring renal parenchymal hypoxia and diffusion-weighted imaging for assessing restricted Brownian diffusion with decreased apparent diffusion coefficient (ADC) in fibrotic AKI kidney have been reported^10, 13^. Liang *et al*. advocated that intravoxel incoherent motion could further permit separate quantification of diffusion and perfusion for evaluating of renal pathophysiological process in contrast-induced AKI^14^. Pohlmann *et al*. proposed the usage of a fast continuous T2*/T2 mapping which is sensitive to blood oxygenation and useful for monitoring *in vivo* changes of initial IRI phase in a unilateral AKI model by using 9.4-tesla small animal MRI system^15^. Although T1 and T2 mapping has been well established for the quantification of myocardial edema or fibrosis in cardiomyopathy and myocardial infarction^16, 17^, investigations on the applications of T1 and T2 mapping in the assessment of AKI are limited^18, 19^. On the other hand, most experiments used a unilateral renal IRI model to attain a final outcome with renal atrophy, and the histopathological examinations were limited to relatively chronic changes^10–12, 18, 19^. In human study, AKI-related histopathological evaluations relied on limited biopsied specimens^13^. However, in reality, AKI caused by critical condition with compromised hemodynamic status, sepsis, or shock because of systemic insult, usually involves both kidneys. In renal injury or inflammation, monocyte chemoattractant protein-1 (MCP-1) is a key early mediator with potent chemoattraction facilitating monocytes and macrophages migration into an inflammatory site^20, 21^. Rapid expression of MCP-1 protein in the tubular epithelial cells and interstitial substance of the medulla marks hyperacute AKI changes before inflammatory cell infiltration^22–24^. Whether ADC, T1, and T2 mapping can be applied for detecting hyperacute changes of AKI has not been well addressed. The purpose of the present study was to investigate the hyperacute and acute changes in ADC, T1, and T2 mapping after severe bilateral renal IRI with longitudinal and cross-sectional assessments, and immunohistochemical (IHC) correlations in a rat model.

## Results

### Serial Changes of Serum Creatinine Levels

**Figure 1:**
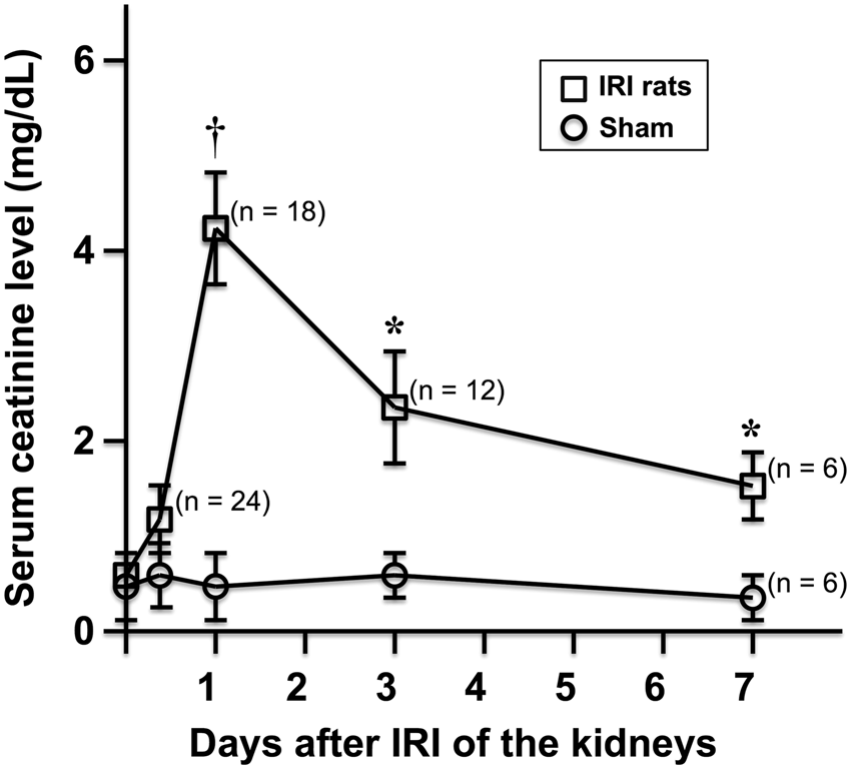
Changes of serum creatinine levels over time. After severe IRI of both kidneys, a trend of elevation of serum creatinine level was noted at 6 hours (P = 0.052) but afterward, significantly elevated serum creatinine levels were noted at all time points as compared with the sham control (n = 6), with peak level at day 1 († indicates P < 0.001, * indicates P < 0.05).

After clamping of bilateral renal pedicles for 60 minutes, there was non-significant elevation of serum creatinine level at 6 hours after IRI as compared with sham control. Subsequently, a rapid upsurge of serum creatinine to peak level occurred at day 1 (P < 0.001) followed by gradual decrement at days 3 and 7 (P < 0.05) (Fig. 1).

### Serial Changes of ADC, T1, and T2 Relaxation Times

**Figure 2:**
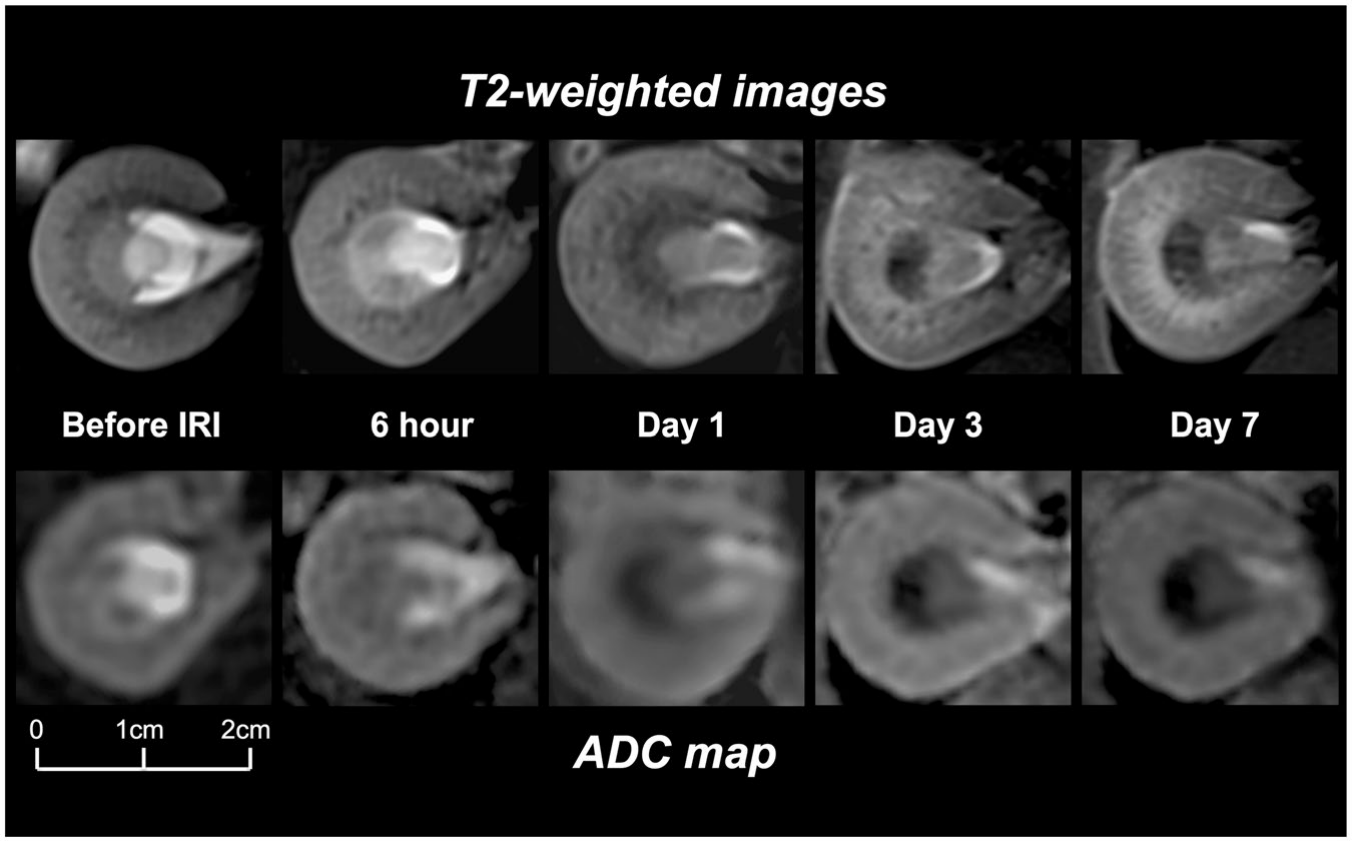
Examples of longitudinal changes of T2-weighted images and ADC maps before and after IRI at 6 hours, days 1, 3, and 7 in a rat kidney. Note subtle changes of the renal cortex, gradual hyperintense changes of OSOM, and progressive hypointense changes of ISOM over time on T2-weighted images. The progression of fluid restriction of ISOM is apparent on ADC maps.

The ADC_avg_, ADC_high_, T1, and T2 values of the cortex, OSOM, and ISOM at different time points after bilateral severe IRI and comparisons are summarized in Table 1 and illustrated in Fig. 2. There were no significant differences of baseline ADC_avg_, ADC_high_, T1, and T2 values between the rats in 5 groups. When compared with the baseline ADC_avg_, the baseline ADC_high_ values were significantly lower in the cortex while the OSOM and ISOM showed non-significant decrement. For IRI rats at different time points, all ADC_high_ values in the three layers exhibited non-significant decrement when compared with the corresponding ADC_avg_.

**Table 1:** Mean apparent diffusion coefficient (ADC), T1-values and T2-valves changes of the rats before and after acute ischemic/reperfusion injury of the kidneys at different time points.

	ADC_avg_	P^§^	ADC_high_	P^§^	ADC_avg_ vs ADC_high_	T1-value (msec)	P^§^	T2-value (msec)	P^§^
	(mean ± SD)		(mean ± SD)		P^#^	(mean ± SD)		(mean ± SD)	
**Cortex**									
Baseline (Gps 1–5)	1.78 ± 0.13–1.88 ± 0.11		1.69 ± 0.22–1.74 ± 0.14		0.045	1462 ± 51–1517 ± 79		84.2 ± 4.5–86.8 ± 3.2	
6 hrs (Gps 1 + 2)	1.77 ± 0.21	0.071	1.65 ± 0.18	0.077	0.067	1520 ± 100	0.063	87.3 ± 5.9	0.294
day 1 (Gps 1 + 3)	1.62 ± 012	0.002	1.56 ± 0.13	0.025	0.375	1648 ± 69	0.012	92.8 ± 3.8	0.002
day 3 (Gps 1 + 4)	1.64 ± 0.09	0.002	1.59 ± 0.18	0.038	0.225	1609 ± 47	0.025	93.9 ± 3.9	0.013
day 7 (Gp 1)	1.68 ± 0.09	0.027	1.64 ± 0.08	0.047	0.415	1556 ± 66	0.263	87.3 ± 3.9	0.372
**OSOM**									
Baseline (Gps 1–5)	1.60 ± 0.11–1.64 ± 0.15		1.57 ± 0.08–1.60 ± 0.13		0.187	1406 ± 48–1434 ± 76		79.3 ± 4.2–82.3 ± 7.1	
6 hrs (Gps 1 + 2)	1.55 ± 0.06	0.023	1.52 ± 0.11	0.043	0.135	1441 ± 78	0.085	84.2 ± 4.2	0.058
day 1 (Gps 1 + 3)	1.52 ± 0.05	0.006	1.49 ± 0.14	0.013	0.416	1551 ± 67	0.005	95.8 ± 5.1	0.014
day 3 (Gps 1 + 4)	1.53 ± 0.10	0.023	1.50 ± 0.12	0.031	0.292	1501 ± 58	0.041	101.0 ± 3.9	0.002
day 7 (Gp 1)	1.56 ± 0.10	0.048	1.54 ± 0.16	0.043	0.283	1491 ± 42	0.028	100.7 ± 8.9	0.028
**ISOM**									
Baseline(Gps 1–5)	1.56 ± 0.15–1.60 ± 0.08		1.53 ± 0.19–1.58 ± 0.07		0.154	1833 ± 58–1880 ± 89		94.1 ± 1.4–95.9 ± 3.4	
6 hrs (Gps 1 + 2)	1.40 ± 0.05	0.002	1.36 ± 0.21	0.004	0.512	1880 ± 141	0.562	96.5 ± 4.6	0.256
day 1 (Gps 1 + 3)	1.20 ± 0.02	0.002	1.17 ± 0.12	0.003	0.457	1831 ± 121	0.273	87.2 ± 2.6	0.002
day 3 (Gps 1 + 4)	1.11 ± 0.11	0.012	1.08 ± 0.15	0.009	0.373	1726 ± 33	0.002	79.2 ± 3.2	0.002
day 7 (Gp 1)	1.03 ± 0.07	0.028	1.01 ± 0.17	0.021	0.662	1672 ± 59	0.028	74.9 ± 2.1	0.017

ADC = apparent diffusion coefficient (x10^−3^ mm^2^/sec), ADC_avg = _average ADC of all b values, ADC_high_ = ADC of high b values, Gp = group, hrs = hours, OM = outer medulla, OS = outer stripe, IS = inner stripe, Gp 5 = sham control (baseline MRI then sacrificed at day 7), Wicoxon signed-rank test (^§^compared with its own baseline values and ^#^compared ADC_avg_ with ADC_high_, significance at P < 0.05).

For the cortex, the ADCs, T1, and T2 values at 6 hours after IRI showed no significant differences as compared with baseline values. At days 1 and 3, the cortex exhibited significantly lower ADCs and significantly higher T1 and T2 values than baseline values. At day 7, ADCs were still significantly decreased, whereas T1 and T2 values reduced with no significant differences from baseline values. Of note, the ADCs of OSOM and ISOM at 6 hours after IRI were significantly lower than baseline values but T1 and T2 values showed non-significant increment. At days 1, 3, and 7, OSOM demonstrated significantly lower ADC and significantly higher T1 and T2 values than baseline values. However, compared with day 3, there was minimal recovery with mild increment of ADCs and decrement of T1 and T2 values at day 7. Except for non-significant decrement of T1 values at day 1, ISOM displayed significantly decreasing ADCs, T1, and T2 values from day 1 to day 7.

### Histopathological and Immunohistochemical Studies

The serial changes of the percentage area with positive MCP1 staining, the number of CD68+ cells, the percentage areas with tubular casts and fibrosis in the cortex, OSOM, and ISOM are illustrated in Fig, 3. At 6 hours or hyperacute phase after severe IRI of both kidneys, only MCP-1 study revealed significantly increased positive staining areas in OSOM and ISOM as compared with the sham (Fig. 4). On the other hand, only non-significant mild increment in number of CD68+ cells, areas with tubular cast formation, and fibrosis were detected.

**Figure 3:**
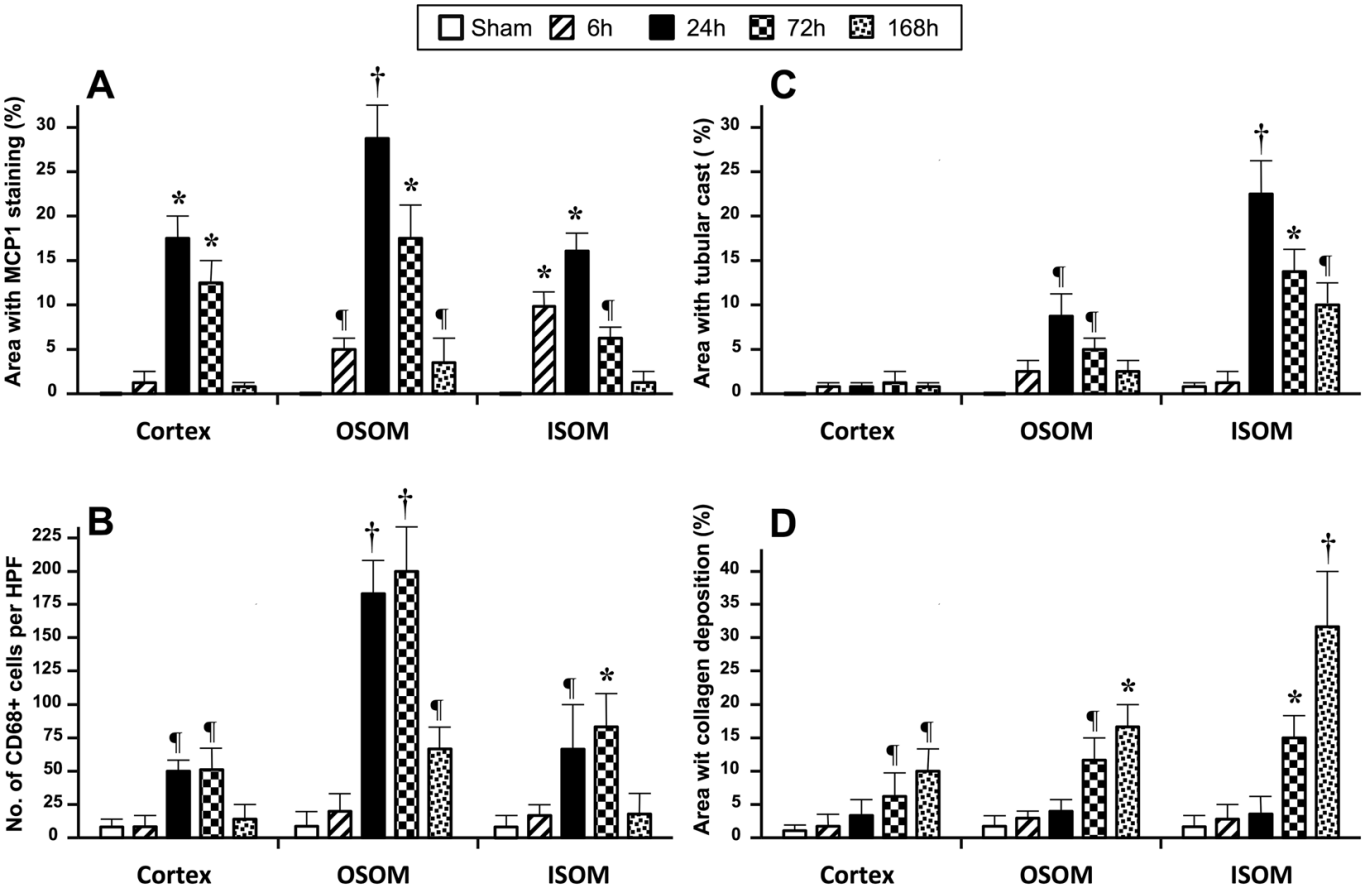
Bar chart illustrations of changes in MCP-1, CD68+ cells, tubular cast formation, and collagen deposition (sirius red) in immunohistochemical studies at different time points among the cortex, OSOM, and ISOM as compared with the sham control (¶ indicates P < 0.05, * indicates P < 0.01, and † indicates P < 0.001). (**A**) Percentage changes in areas with positive MCP-1 staining. OSOM and ISOM showed significantly larger areas with MCP-1 staining at 6 hours. All three zones at days 1 and 3 and OSOM at day 7 showed significant increment in areas with MCP-1 staining increases, with peak level at day 1. (**B**) Changes in number of CD68+ cells. All three zones had significant increases in the number of CD68+ cells at days 1 and 3, and OSOM at day 7 with peak level at day 3, especially prominent in OSOM. (**C**) Percentage changes in areas with tubular cast formation. The renal cortex showed no significant differences at all time points. OSOM and ISOM showed significantly more tubular casts at days 1 and 3, and days 1, 3, and 7 respectively with peak level at day 1. (**D**) Percentage changes in collagen deposition. As compared with the sham control, all three zones showed no significant differences in collagen deposition at 6 hours and day 1 after IRI. At days 3 and 7, all three zones harbored significantly more collagen with peak level at day 7, while ISOM was more severely affected.

**Figure 4:**
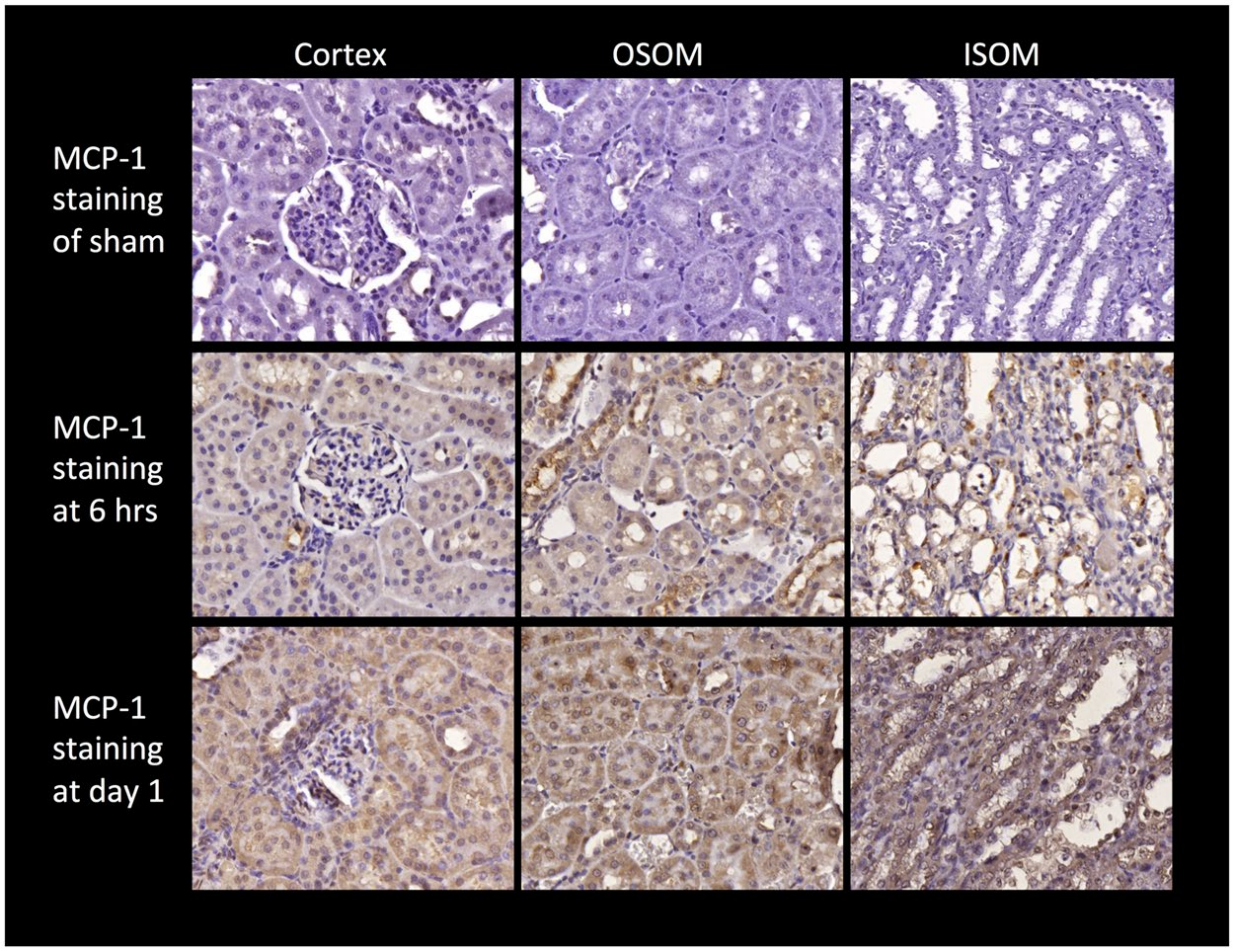
Immunohistochemical studies of MCP-1 in the cortex, OSOM, and ISOM of the sham, the rats at 6 hours and day 1 after IRI. MCP-1 staining (brown color) can barely be seen in the sham control in all three zones (top row). Hyperacute changes after IRI with obvious MCP-1 staining can be detected in OSOM and ISOM at 6 hours (middle row). By day 1, all three zones exhibit prolific MCP-1 staining (bottom row).

As compared with the sham, significantly increased MCP-1 staining was noted in all three zones at days 1 and 3 and OSOM at day 7, with peak level at day 1 followed by gradual decrement. A significant increased number of CD68+ cells was also noted in all three zones at days 1 and 3, and OSOM at day 7 with peak level at day 3. For tubular cast formation, the renal cortex showed no significant differences at all time points. As compared with the sham, OSOM and ISOM harbored significantly more tubular casts from days 1 and 3, and days 1, 3, and 7, respectively, with peak level at day 1. For collagen deposition or degree of fibrosis, there were no significant differences between sham control and the kidneys at 6 hours and day 1 after IRI. However, all three zones showed significantly more collagen deposition at days 3 and 7 than the sham control with peak level at day 7, whilst ISOM was more severely affected.

### Relationships between MR Parameters and Immunohistochemical Changes

**Figure 5:**
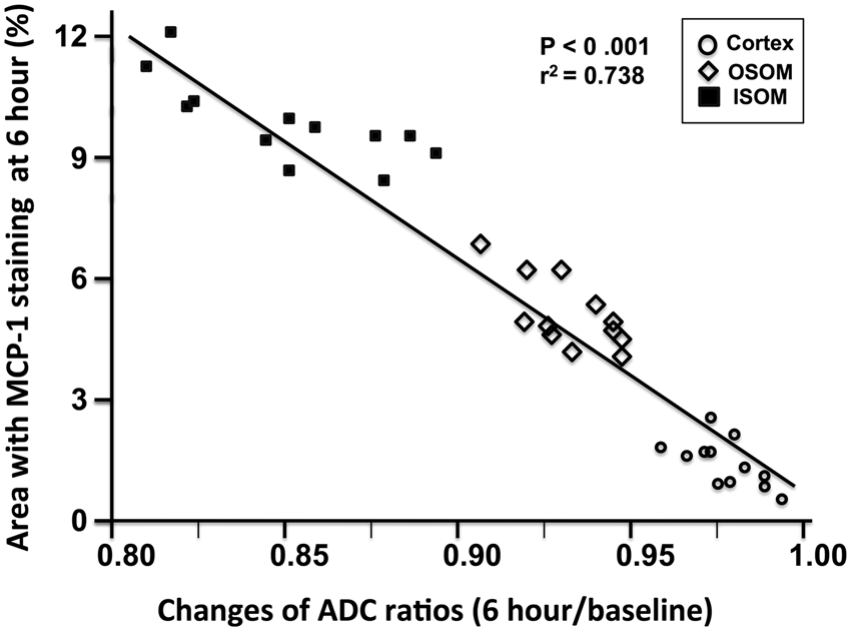
Simple regression analysis of the rats (twelve kidneys) in Group 2 sacrificed at 6 hours after the MRI studies shows a significant inverse correlation between the percentage area with MCP-1 staining and 6-hour/baseline ADC ratios (P < 0.001, r^2^ = 0.738).

At 6 hours after IRI, only the ADC values of the renal OSOM and ISOM were found to exhibit significant decrement as compared to baseline values. Among six rats in Group 2 sacrificed after a 6-hour MRI follow-up, only MCP-1 study revealed significant increment in OSOM and ISOM. A simple regression analysis revealed strong inverse correlation between the percentage area with MCP-1 staining at 6 hours and 6-hour/baseline ADC ratios (P < 0.001, r^2^ = 0.738) (Fig. 5). However, no other significant correlations between ADCs, T1 or T2 values and histopathological or immunohistochemical parameters could be identified, possibly owing to complex IRI changes and multiple factors might exhibit simultaneous influences on MRI changes after early MCP-1 proliferation.

At days 1 and 3, rapid upsurges of MCP-1 staining in all three zones were followed by consequential significant increased CD68+ cells with peak levels at day 3. At days 1 and 3, significant decrement of ADC in all three zones at days 1 and 3 plausibly ascribed to fluid restriction related to markedly increased MCP-1 protein and CD68+ cell infiltration. Furthermore, significantly increased T1 and T2 values in the cortex and OSOM were compatible with prominent inflammatory edema induced by monocytes/macrophages-related inflammatory responses. At day 7, decreased ADC values concurred with increased fibrosis in the cortex and marked reduced CD68+ cells indicated considerable recovery from inflammation resulting in decrement of T1 and T2 values. In contrast, OSOM harbored less pronounced improvement by day 7 with persistent significantly lower ADC and higher T1and T2 values, which might represent a combined effect of increased fibrosis, less MCP-1 staining, and detectable remaining inflammatory edema as reflected by residual teeming CD68+ cells. It is noteworthy that there were progressive decrement and significantly lower ADC, T1, and T2 values of ISOM from day 1 to day 7 after IRI concurred with markedly increased tubular cast formation with peak level at day 1 and significantly increased collagen deposition from day 3 to day 7.

## Discussion

Despite the application of functional MRI in assessing IRI in animal or clinical studies that have been reported^10–15, 18, 19^, to our knowledge, this is the first study with longitudinal and cross-sectional MRI evaluations of the early changes of ADC, T1, and T2 values in the renal cortex, OSOM, and ISOM with thorough histopathologic and IHC correlations after severe bilateral IRI. Four histopathologic or IHC changes were assessed in detail in three different zones separately at various time points to evaluate the hyperacute changes with MCP-1 immunostaining, acute changes by severities of inflammation cell infiltration (CD68+ cells) and tubular cast formation, and late phase changes by degree of fibrosis (collagen deposition).

In contrast to unilateral renal IRI animal model, both renal pedicles were clamped simultaneously in the present study and thus the effect of contralateral kidney compensation could be eliminated. Consistent with prior studies, even after severe IRI of both kidneys, our results also revealed that the serum creatinine changes were less apparent initially for several hours, while subsequent rapid deterioration of renal function occurred in 24 hours and such a lag time between renal injury and the increase in the serum creatinine level can lead to missed early therapeutic opportunities^6, 24, 25^.

Diffusion-weighted imaging and ADC measurement can offer quantitative information on the microstructure and water motion on a molecular level^10, 13, 14, 18, 19^. Although ADC measurement with conventional monoexponential model delivers signals from both capillary perfusion and extracellular diffusion, measurement of ADC with *b*-values > 400 sec/mm^2^ primarily referring to molecular water diffusion for blood perfusion signals are substantially suppressed by the large diffusion gradients^13, 14, 26, 27^. In the present study, 7 b-valves (0, 50, 150, 450, 650, 850, and 1000 sec/mm^2^) were used for calculating ADC_avg_, and the gradients were applied in three orthogonal directions and subsequently averaged so that the effects of diffusion anisotropy could be eliminated^26, 27^. In addition, ADC_high_ with 4 high b-values (450, 650, 850, and 1000 sec/mm^2^) were also calculated to assess the influence of perfusion. We found that the baseline ADC_high_ value of the cortex was significantly lower than the baseline ADC_avg_ while the OSOM and ISOM showed non-significant decrement, indicating affluent cortical perfusion can affect true diffusion measurement. After bilateral IRI, all ADC_high_ values exhibited non-significant decrement when compared with the corresponding ADC_avg_, suggesting that perfusion effect on ADC measurement after IRI was attenuated in the cortex while OSOM and ISOM were less affected. On the other hand, our results disclosed that the both ADC_avg_ and ADC_high_ values of the renal OSOM and ISOM were significantly lower than baseline values at 6 hours after IRI. MCP-1 is a potent chemokine with copious early protein expression in the renal tubules that occur within several hours after IRI^20–23^. Our study also revealed a strong inverse correlation between increased MCP-1 immunostaining at 6 hours and decreased 6-hour/baseline ADC ratios (P < 0.001, r^2^ = 0.738). This might indicate that the changes of ADC in OSOM and ISOM are related to early alteration of cellular content with increased MCP-1 protein in the secreting cells, and thus can be an early biomarker denoting hyperacute microstructural changes of AKI after IRI. Increased T1 and T2 relaxation times are correlated with a greater content of tissue water or inflammatory edema^18, 19, 28, 29^. The lack of significant changes of T1 and T2 values in the cortex at 6 hours after IRI could imply the presence of a lag time before the inflammatory cells infiltration and renal tissue edema become prominent.

In contradistinction to OSOM and ISOM, the present study demonstrates that the renal cortex exhibited no significant changes in ADC, T1, and T2 values at 6 hours. The findings can be attributed to the perfusion difference of the renal cortex and medulla^10, 30^. The degree of perfusion impairment measured using arterial spin labeling has been reported to be related to kidney volume loss, the severity of renal tissue alterations, and the impairment of renal function^10, 30, 31^. As the cortex receives majority (90–94%) of the renal blood flow, whereas only a minor part (5%) is supplied to the medulla, the renal cortex is less susceptible to hypoxia and hypoperfusion^31, 32^. Our study further demonstrates that even though there were rapid upsurges at days 1 and 3, MCP-1 staining and CD68+ cells were markedly decreased in the cortex at day 7 indicating regressive changes of inflammation, whereas the tubular cast formation in the cortex was not apparent at all time points. In line with IHC findings, MRI showed significant increases in T1 and T2 values at days 1 and 3 but no significant differences from baseline values at day 7. On the other hand, our results also revealed that significantly reduced renal cortical ADC values at days 1 and 3 concurred with increased MCP-1 staining and inflammatory cellularity, whereas reduced ADC at day 7 concurred with increased fibrosis on IHC studies.

Physiologically, the renal medulla is vulnerable to hypoxic or ischemic insults ascribed to low blood flow, limited oxygen supply, and heavy oxygen demand for reabsorption of sodium^8, 10, 32^. The kinetics of leukocytes infiltration in the kidney after severe IRI have been described with pronounced tubular necrosis during the first 12 to 24 hours followed by prominent monocytes/macrophage infiltration in the medulla, but only a low number of neutrophils were noted^21, 33^. Histologically, OSOM is composed of pars recta or thick descending and thick ascending ducts whilst ISOM is composed of thin descending and thick ascending ducts. The present study with longitudinal and cross-sectional assessments revealed different manifestations of OSOM and ISOM after IRI.

At days 1 and 3, both OSOM and ISOM exhibited significantly increased MCP-1 staining and inflammatory cell infiltration, more profuse in OSOM, and increased tubular cast formation, more copious in ISOM. While increased MCP-1 secreting cells and CD68+ cell could lead to reduced ADC values, pronounced monocytes/macrophages-induced inflammatory responses with tissue edema could cause early significant elevation of T1 and T2 values in OSOM at day 1. At day 7, OSOM harbored mild recovery with increased fibrosis, less MCP-1 staining, residual inflammatory cell infiltration, and detectable remaining inflammatory edema. A combined effect of such complex changes presented with persistently reduced ADC values and increased T1and T2 values. For ISOM, regardless of initial proliferation of MCP-1 secreting and CD68+ cells, overwhelming tubular casts with proteinaceous or waxy content and increasing fibrogenesis from day 1 to day 7 could cause fluid restriction with reduced ADC values and might out-weigh the inflammatory changes leading to decreased T1 and T2 relaxation times.

This study has limitations. First, the study sample size was relatively small. However, significant differences were shown between different groups in different zones at different time points by longitudinal and cross-sectional assessments with IHC and histological correlations. Second, only MCP-1 was assessed for including all available biomarkers deemed impossible while MCP-1 is crucial for stimulating monocytes/macrophages migration^4, 5, 24, 25^. Third, identifying a specific factor for ADC changes was difficult for rather than a sole mechanism of renal fibrosis^13, 27^, hypercellularity^34^, acute inflammation^26^, and neoplasm^35^ may also lead to ADC changes. We believe that ADC changes in the cortex, OSOM and ISOM in AKI is a summation effect of variable degrees of microstructural changes, inflammatory cell infiltration, inflammation edema, tubular necrosis, cast formation, and collagen deposition at different time points. Fourth, we presume that elevation of T1 and T2 values was ascribed to tissue edema or increased water content as an inflammatory response potentiate via cytokines and toxic mediators produced by monocyte/macrophage^21, 28, 29^.

In conclusion, markedly reduced ADCs in OSOM and ISOM can be used as a non-invasive biomarker denoting hyperacute change of AKI after IRI. ADC, T1, and T2 values are useful for assessing AKI with variable changes in different layers of the kidneys depending on underlying microstructural and histopathological changes at different time points.

## Materials and Methods

### Ethics Statement

The *Institutional Committee on Animal Care, Use and Research* approved the experiment (affidavit number: 2013110102). All applicable institutional and/or national guidelines for the care and use of animals were followed. Pathogen-free, adult male Sprague-Dawley (SD) rats weighing 400–450 g (BIOLASCO TAIWAN CO, LTD) were reared in pathogen-free animal facilities (24 °C ± 1, 55% ± 10 humidity) with water and a standard diet provided ad libitum.

### Renal Ischemic-Reperfusion Injury and Animal Grouping

The SD rats were anesthetized with isoflurane. After median laparotomy and blunt dissection of renal pedicles, non-traumatic vascular clamps were applied to bilateral renal pedicles simultaneously for 60 minutes. After the clamps were removed, reperfusion was confirmed visually. Before closure of the abdomen, the intraperitoneal air and fluid were aspirated out as much as possible. A total of 29 rats received IRI induction and five rats died on day 2 to day 5. Twenty-four survived rats were randomized as four groups. Six rats (Group 1) underwent longitudinal MRI examinations before IRI and after IRI at 6 hours, days 1, 3, and 7, and were sacrificed on day 7 after the MRI study. The 18 remaining rats were sacrificed after MRI studies at 6 hours (Group 2, n = 6), day 1 (Group 3, n = 6) and day 3 (Group 4, n = 6) for cross-sectional assessments. In the present study, we defined the hyperacute changes as the MRI, histopathological and immunohistochemical changes occurred at 6 hours after IRI. A sham-operated group (Group 5, n = 6) underwent a baseline MRI examination before a blunt renal pedicle dissection without clamping, and the rats were sacrificed at day 7 after blood sampling. Blood samplings for the assessment of serum creatinine levels were performed before and after the IRI at 6 hours, days 1, 3, and 7. After 1 mL of blood was drawn from the tail artery and the sample was centrifuged (3000 rpm) for 45 sec. Then the serum (approximate 0.5 mL) was collected and stored in −20 °C.

### MR Imaging

All MR examinations were performed with a 3.0-T MR imager (MAGNETOM^®^ Skyra, Siemens Healthcare, Erlangen, Germany) with a 16-channel wrist coil. After adequate anesthesia to keep shallow breathing, the rat was placed in a supine position in a plastic holder. The imaging parameters are described in Table 2. After initial low-resolution localization images, T2-weighted images with anatomic details encompassing both kidneys were obtained. A diffusion-weighted imaging was performed with 7 b valves (0, 50, 150, 450, 650, 850, and 1000 sec/mm^2^). Motion-sensitive gradients were applied in three orthogonal directions and then averaged to eliminate the anisotropic effects. For quantification of T1 and T2 relaxation times of the kidney, T1 and T2 mapping was done with fast low-angle shot sequence (flip angles of 5° and 26°) and multiecho spin-echo sequence (echo times: 13.8, 27.6, 41.4, 55.2, and 69 msec), respectively.

**Table 2:** Sequence parameters for renal 3.0-T MR imaging in rats with acute ischemic/reperfusion kidney injury.

	T2WI	DWI (ADC)	T1-Map	T2-Map
Sequence	TSE	SE-EPI	FLASH	SE
Repetition time (msec)	3000	5000	15	1360
Echo time (msec)	73	82	2.42	13.8, 27.6, 41.4 55.2, 69
Flip angle (degree)	150	NA	5, 26	180
Matrix	256 × 256	128 × 128	256 × 256	256 × 256
Field of view (cm^2^)	14	14	14	14
Section thickness (mm)	3	3	3	3
Intersection gap (mm)	0.3	0.3	0.3	0.3
Number of excitation	2	1	1	1
Number of slices	12	12	12	12
b-value used (sec/mm^2^)	NA	0, 50, 150, 450, 650, 850, 1000	NA	NA

T2WI = T2-weighted image, DWI (ADC) = diffusion-weighted imaging (apparent diffusion coefficient), TSE = turbo spin-echo, EPI = echo-planar imaging, FLASH = Fast low angle shot, SE = spin-echo, TR = repetition time, TE = echo time, NA = not applicable.

**Figure 6:**
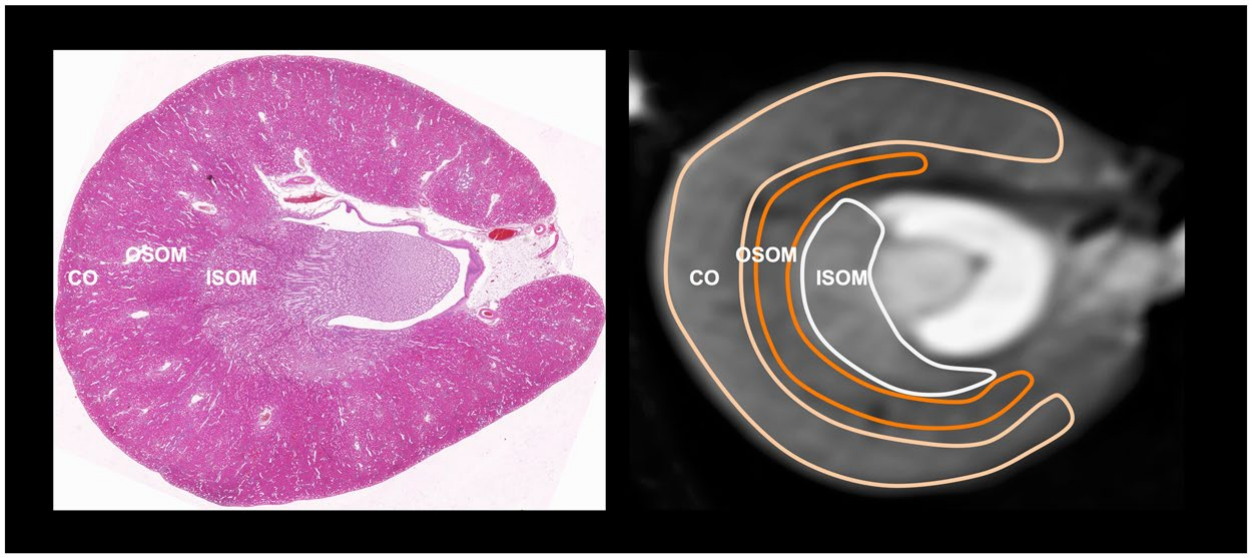
Axial histological section at kidney (left) and corresponding T2-weighted image (right) at the central part of the kidney illustrate the placement of regions of interest along the boundaries of the anatomic zones of the renal cortex (CO) outer strip of the outer medulla (OSOM) and inner stripe of the outer medulla (ISOM). The ROI outlines were copied and pasted on the ADC, T1, and T2 maps for the measurements of ADC values, and T1and T2 relaxation times.

The ADC, T1, and T2 maps were analyzed in consensus by two experienced readers who were blinded to the animal group identity on a workstation (Siemens, Syngo medical software). The rat kidney is unipapillary with a cup-shaped renal cortex surrounds the renal medulla that can be divided into inner medulla (composed of thin descending and thin ascending ducts) and outer medulla (with the outer stripe composed of pars recta or thick descending and thick ascending ducts, and the inner stripe composed of thin descending and thick ascending ducts). Regions of interest (ROIs) were placed manually into the cortex, the outer stripe of outer medulla (OSOM), and the inner stripe of outer medulla (ISOM) of both kidneys on T2-weighted images (Fig. 6). The same ROIs were then copied and pasted on the ADC, T1, and T2 maps for the determination of ADC, T1, and T2 values, respectively. In order to assess the influence of perfusion on ADC measurement, in addition to average ADC (ADC_avg_) of all 7 b values (b = 0, 50, 150, 450, 650, 850, and 1000 sec/mm^2^), a separate analysis of ADC for the high b values (ADC_high_, b = 450, 650, 850, and 1000 sec/mm^2^) was also performed. For each kidney, two central axial slices were selected for the measurements and the values obtained were averaged.

### Histopathological and Immunohistochemical Studies

The kidney specimens from all the animals were fixed in 10% neutral-buffered formalin and then embedded in paraffin. The paraffin-fixed kidney specimens were axially sectioned at 4 μm for hematoxylin-eosin (H and E) staining and histopathological evaluation. For IHC study of MCP-1 and CD68+ cells, after 4-μm paraffin-embedded axial sections were deparaffinized and rehydrated with xylene, ethanol, and wash buffer, the slides were treated with rabbit polyclonal anti-MCP-1 and anti-CD68 antibodies (Abcam) respectively. Endogenous peroxidase was quenched with 0.3% hydrogen peroxide for 20 minutes. The secondary antibody for immunostaining was biotinylated with anti-mouse immunoglobulins (Abcam) and an avidin-biotin horseradish peroxidase kit (mouse and rabbit specific HRP/DAB [ABC] detection IHC kit, Abcam) was applied. Visualization of peroxidase localization was accomplished by using diaminobenzidine (DAB)-hydrogen peroxide substrate to give a brown color. Finally, the tissue sections were counterstained with hematoxylin, dehydrated, and mounted for assessment. For a negative control, normal rabbit immunoglobulin was used as a primary antibody (Abcam). IHC study by using sirius red (Direct Red 80, Sigma-Aldrich) was performed for assessing collagen deposition (an indicator of degree of fibrosis). After the slices were deparaffinized and rehydrated, the slides were stained with picro-sirius red followed by washing with acidified water. Then excessive water was physically removed by vigorous shaking. Finally, the slides were dehydrated with 100% ethanol, cleared, and mounted in a resinous medium.

Six rats (12 kidneys) were available in each group and for each kidney, two central axial tissue sections were selected for each of the IHC studies (MCP-1, CD68+ cells and sirius red) and histopathological analysis of tubular casts. After appropriate staining, the whole slides were scanned with the Panoramic MIDI high-resolution digital scanner (3DHISTECH Ltd., Budapest, Hungary). Post-processing was performed with Panoramic Viewer 1.15 (3DHISTECH Ltd., Budapest, Hungary). In each scanned tissue section, five randomly chosen areas (300 × 400 μm^2^, corresponding to high-power-field 500x on light microscopy) in each zone (cortex, OSOM, and ISOM) were recorded and captured as high resolution TIF files. Afterward, the captured images were assessed with a 2D-image analysis software (Image-Pro Plus, Media Cybernetics Inc., Rockville, MD). After spatial area calibration of the captured image as 300 × 400 μm^2^, a user-assigned color (bright green in the present study) was selected for tagging of ROI for the distribution analysis. For defining the ROI, the caliper in measure tool was picked and the caliper icon was placed on the scanned image with positive staining (MCP-1, brown color; sirius red, red color, CD68+ cells, brown color, or tubular cast, pink color). The optical density of a selected point was used as a reference for auto-sorting all the areas with the same density of staining. Finally, color-tagging of ROI and measurement of areas (or cell counting) were achieved by automatic measurement tools. The mean percentages of ROI with positive staining of MCP-1, serious red, and tubular casts, and mean number of CD68+ cells in each zone of each kidney were determined by summing all numbers divided by 10 (for a total of 10 high-power-fields, 2 sections were assessed).

### Statistical Analysis

The data are expressed as mean ± standard deviation unless otherwise stated. The serum creatinine levels of IRI rats were compared to sham control at different time points using Mann-Whitney U test. Comparisons of the ADC_avg_, ADC_high_, T1, and T2 values of the three zones (cortex, OSOM, ISOM) at 6 hours, days 1, 3, and 7 after IRI with its own baseline values as well as comparisons between ADC_avg_ and ADC_high_ values were performed using the Wilcoxon signed-rank test. Comparisons of the percentages of areas with positive staining of MCP-1, tubular cast, and serious red, and number of CD68+ cells in three zones at different time points with sham control were made by Mann-Whitney U test. The relationship between the percentage area with positive MCP-1 staining at 6 hours and 6-hour/baseline ADC ratio was assessed with a simple linear regression analysis. The statistical analyses were performed using the SYSTAT software (SPSS for Windows, version 13; IL, USA), and a *P* value < 0.05 was considered statistically significant.
